# Characterization of technical skill progress in a standardized rabbit model for training in laparoscopic duodenal atresia repair

**DOI:** 10.1007/s00464-021-08530-x

**Published:** 2021-05-17

**Authors:** Péter Etlinger, Catarina Barroso, Alice Miranda, João Moreira Pinto, Ruben Lamas-Pinheiro, Hélder Ferreira, Pedro Leão, Tamás Kovács, László Juhász, László Sasi Szabó, András Farkas, Péter Vajda, Attila Kálmán, Tibor Géczi, Zsolt Simonka, Tamás Cserni, Miklós Nógrády, Gergely H. Fodor, Andrea Szabó, Jorge Correia-Pinto

**Affiliations:** 1 Life and Health Sciences Research Institute (ICVS), School of Medicine, University of Minho, Braga, Portugal; 2 ICVS/3B’s-PT Government Associate Laboratory, Braga/Guimarães, Portugal; 3 Division of Pediatric Surgery, Department of Pediatrics, University of Szeged, Korányi fasor 14-15, 6720 Szeged, Hungary; 4 Department of Pediatric Surgery, Hospital de Braga, Braga, Portugal; 5 Institute of Surgical Research, University of Szeged, Szeged, Hungary; 6 Pediatric Surgery, Hospital-Escola da Universidade Fernando Pessoa, Gondomar, Portugal; 7 EpiUnit, Instituto de Saúde Pública da Universidade do Porto, Porto, Portugal; 8 Minimally Invasive Gynecology Department, Centro Hospitalar Universitario do Porto EPE-Centro Materno Infantil do Norte, Porto, Portugal; 9 Division of Pediatric Surgery, Department of Pediatrics, University of Debrecen, Debrecen, Hungary; 10 Division of Pediatric Surgery, Department of Pediatrics, University of Pécs, Pécs, Hungary; 11 Division of Pediatric Surgery, Department of Pediatrics No. I, Semmelweis University, Budapest, Hungary; 12 Department of Surgery, University of Szeged, Szeged, Hungary; 13 Department of Paediatric Urology, Royal Manchester Children’s Hospital, Manchester, UK; 14 Department of Gynecology, Kiskunhalas Teaching Hospital, University of Szeged, Szeged, Hungary; 15 Department of Medical Physics and Informatics, University of Szeged, Szeged, Hungary

**Keywords:** Pediatric surgery, Laparoscopy, Diamond-shaped anastomosis, GOALS score

## Abstract

**Background:**

Laboratory skills training is an essential step before conducting minimally invasive surgery in clinical practice. Our main aim was to develop an animal model for training in clinically highly challenging laparoscopic duodenal atresia repair that could be useful in establishing a minimum number of repetitions to indicate safe performance of similar interventions on humans.

**Materials and methods:**

A rabbit model of laparoscopic duodenum atresia surgery involving a diamond-shaped duodeno-duodenostomy was designed. This approach was tested in two groups of surgeons: in a beginner group without any previous clinical laparoscopic experience (but having undergone previous standardized dry-lab training, *n* = 8) and in an advanced group comprising pediatric surgery fellows with previous clinical experience of laparoscopy (*n* = 7). Each participant performed eight interventions. Surgical time, expert assessment using the Global Operative Assessment of Laparoscopic Skills (GOALS) score, anastomosis quality (leakage) and results from participant feedback questionnaires were analyzed.

**Results:**

Participants in both groups successfully completed all eight surgeries. The surgical time gradually improved in both groups, but it was typically shorter in the advanced group than in the beginner group. The leakage rate was significantly lower in the advanced group in the first two interventions, and it reached its optimal level after five operations in both groups. The GOALS and participant feedback scores showed gradual increases, evident even after the fifth surgery.

**Conclusions:**

Our data confirm the feasibility of this advanced pediatric laparoscopic model. Surgical time, anastomosis quality, GOALS score and self-assessment parameters adequately quantify technical improvement among the participants. Anastomosis quality reaches its optimal value after the fifth operation even in novice, but uniformly trained surgeons. A minimum number of wet-lab operations can be determined before surgery can be safely conducted in a clinical setting, where the development of further non-technical skills is also required.

The number of advanced endoscopic surgical interventions is continuously increasing worldwide. Nonetheless, pediatric surgery faces special challenges, including the use of special surgical instruments and limited interventions due to a smaller workspace, with the safety of surgical interventions playing a particularly important role with children [1]. Furthermore, pediatric surgery is not typically divided into subspecialties; every pediatric surgeon is therefore expected to be familiar with numerous types of surgical interventions. Duodenual atresia is one of the technically most challenging neonatal laparoscopic interventions. All these facts underline the importance of laboratory training designed to master advanced techniques before carrying them out on pediatric patients [2].

As for training alternatives, high-fidelity models are required to adequately simulate pediatric surgical conditions. Although many simulation-based training methods have been established [2–4] and numerous inanimate solutions have appeared recently [4, 5], the superiority of live anesthetized animal models over *ex vivo*, virtual reality and plastic models has been suggested [6–13]. A key issue here is transferability of laboratory skills to real clinical scenarios. Quantitative assessment can focus on multiple factors, such as (a) duration of the intervention, (b) success of the surgery, (c–d) occurrence of a number of intraoperative complications and their management, or (e) the surgeon’s technical skills and bimanual dexterity. (f) Another important aspect is self-evaluation of the participants’ own performance using standardized criteria [2, 12, 13].

The aim was to define criteria for a novel standardized rabbit model of duodenal atresia (diamond-shaped anastomosis) which could enable training participants to perform the same surgical pediatric intervention in clinical practice. According to our hypothesis, the model is appropriate and sufficiently complex to evaluate advancement and compare development of the technical skills of the trainee groups with different levels of expertise using learning curve-based assessment methods. Furthermore, we hypothesized that a minimal repetition number of diamond-shaped anastomosis surgeries can be defined in this animal model by which threshold values for clinical transferability to complex operations can be recommended.

## Materials and methods

### Participants

The present study was conducted between September 2016 and September 2017 at the Endoscopic Research and Training Laboratory of the Surgical Sciences of the Life and Health Sciences Research Institute in Braga, Portugal. A total of 15 laparoscopic trainees were recruited and allotted into one or the other of two groups. (1) A *beginner group* (*n* = 8) consisted of medical doctors soon after graduation without any previous laparoscopic experience. This group underwent at least twelve hours of laparoscopic training with the same supervisor based on a modified, previously validated inanimate assessment method known as the McGill Inanimate System for Training and Evaluation of Laparoscopic Skills (MISTELS) [14]. An *advanced group* (*n* = 7) comprised pediatric surgery fellows with previous laparoscopic experience of at least 25 human cases (e.g. appendectomy, varicocelectomy or herniorrhaphy). These fellows had not received any standardized and structured, minimally invasive laparoscopy surgery training at this institute.

### Surgical procedure

#### Setup of the test operation

**Fig. 1:**
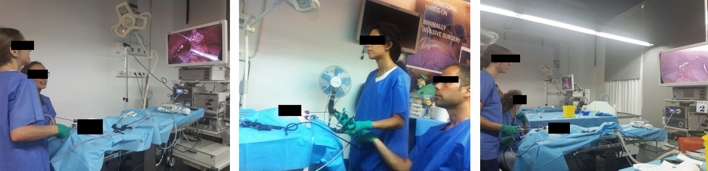
Laparoscopic setup for training

Participants in both groups were invited to perform eight laparoscopic, diamond-shaped bowel anastomosis surgeries using the same surgical technique and steps on anesthetized rabbits (see below). Scheduling of the operations was not pre-determined (it was dependent on participant preferences) with a maximum of two operations per day. The interval between the first and last operations ranged between four and 150 days. All test operations were supervised by two instructors, who had already performed at least 15 surgeries using the same model. Continuous guidance was provided based on drawn schemes and video tutorials of the procedure (Fig. 1).

#### Equipment

Karl Storz laparoscopic equipment and recording devices were used during all of the surgeries. Insufflation was performed with CO_2_ using 6 mmHg pressure and 1.5 L/min gas flow. Surgical instruments included 5-mm telescope 30º and 3-mm instruments (Maryland dissector, bowel grasper, needle holder, anatomical forceps and scissors). 2/0 Prolene thread (Ethicon, Inc., Somerville, NJ, USA) was used for bowel suspension, and 5/0 Prolene (Ethicon, Inc., Somerville, NJ, USA) was used for continuous suture of the anastomoses. Port placement followed the standard method.

#### Animal model

Ethical approval for this study was obtained from the Portuguese General Directorate for Food and Veterinary Affairs (Direção Geral de Alimentação e Veterinária-DGAV 0421/000/000/2017) and the University of Minho Ethics Committee (SECVS 004/2016). *Oryctolagus cuniculus* rabbits weighing 2000–2500 g were used. Anesthesia was achieved using ketamine (35 mg/kg; Ketamidor, Richter Pharma AG, Austria), medetomidine (0.5 mg/kg; Sededorm, VetPharma Animal Health, Spain) and buprenorphine (0.03 mg/kg; Bupaq, Richter Pharma AG, Austria) administered through the ear vein. Every animal underwent a tracheostomy and was ventilated. Animals were sacrificed using pentobarbital (200 mg/kg; Euthasol, Le Vet Beheer B.V., Netherlands) after surgery. In compliance with the 3R principle, more than one surgical intervention (with a maximum of three) was performed per animal.

#### Method of test operation

**Fig. 2:**
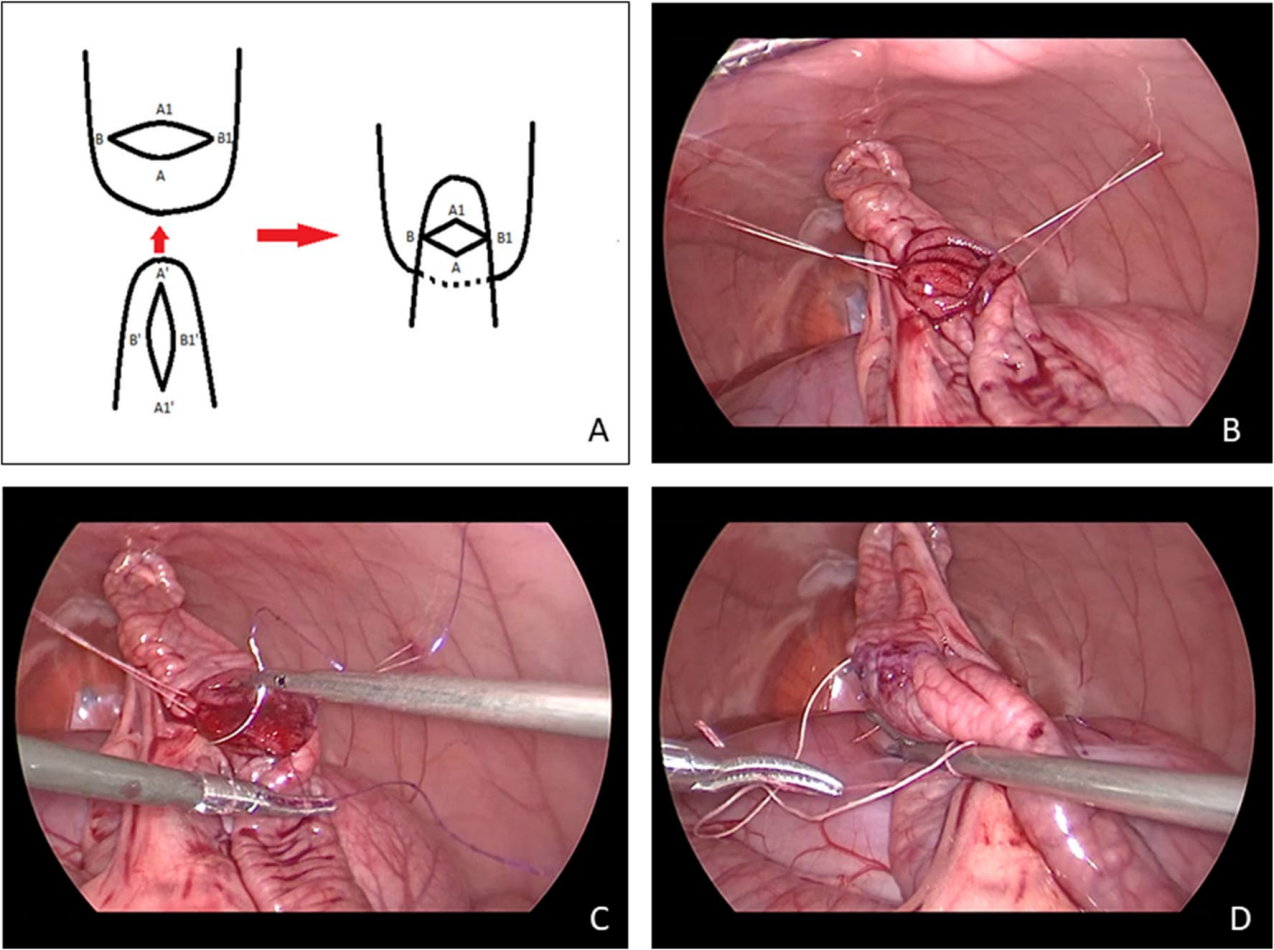
**A** Scheme for diamond-shaped anastomosis (the order of stitches follows reference points A-A′, B-B′, A1-A1′ and B1-B1′, with continuous sutures on the posterior and anterior walls); intraoperative photos: **B** after suspending the bowel; **C** suturing the posterior wall; and **D** placing the last stitch after suturing the anterior wall

An optical port was inserted through the umbilical region of the rabbit followed by symmetrical placement of two working ports bilaterally. A jejunal segment was selected and suspended to the abdominal wall (Fig. 2). A proximal transverse enterotomy and a distal longitudinal one were made in the selected bowel segment to simulate the atretic stumps. This method was established based on preliminary studies with less ideal results involving anastomoses between the stomach and small bowel, between the small bowel and the vermiform appendix and between the gallbladder and the small intestine. Two fixation corner stitches were then placed to unite the proximal and distal stumps according to the method developed by Kimura et al. (Fig. 2A) [15, 16]. After suturing the posterior wall with continuous sutures, the anterior site was approximated in the same manner, followed by tying the stitches at the corners (Fig. 2B−D).

#### Video processing and segmentation

Each video segment of the net anastomosis procedure was saved separately, encoded with randomly generated numbers, shared using secure cloud storage and used for analysis. The duration of the segments was recorded. Each of the eight surgeries per participant was encoded separately. Each segment was assessed by four experts in a randomized, blinded fashion using individual information sheets (containing assessment criteria and video codes, but no information about the identity of the participant or the stage of the learning process).

#### Assessments and parameters examined

Four parameters were used after each surgery:

- Surgical time: The time interval between proximal opening of the bowel segment and the last surgical knot.
- Quality of the anastomosis: luminal passage and macroscopic leakage of the anastomosis were assessed after the animals were sacrificed by pressing the luminal content through the anastomosis.
- Expert evaluation was performed using the modified Global Operative Assessment of Laparoscopic Skills (GOALS) score [17]: video recordings were assessed in a blinded fashion by fellow surgeon experts in laparoscopy using the GOALS score developed by Vassiliou et al. [18] based on certain criteria (see Table 1).
- Participants’ feedback: a 1−5-scale questionnaire was used for the following parameters: (1) working space, (2) workflow, (3) level of self-confidence and (4) level of self-achievement.

**Table 1:** Parameters and GOALS score values used in the study

Parameters	Score		
	1	3	5
Depth perception	Constantly overshoots target, wide swings, slow to correct	Some overshooting or missing of target, but quick to correct	Accurately directs instruments to target in correct plane
Bimanual dexterity	Uses only one hand, ignores nondominant hand, poor coordination between hands	Uses both hands, but does not optimize interaction between hands	Expertly uses both hands in a complementary manner to provide optimal exposure
Efficiency	Uncertain, inefficient efforts; many tentative movements; constantly changing focus or persisting without progress	Slow, but planned movements are reasonably organized	Confident, efficient and safe conduct, maintains focus on task until performance is improved via an alternative approach
Tissue handling	Rough movements, tears tissue, injures adjacent structures, poor grasper control, grasper frequently slips	Handles tissues reasonably well, minor trauma to adjacent tissue (i.e. occasional unnecessary bleeding or slipping of the grasper)	Handles tissues well, applies appropriate traction, negligible injury to adjacent structures
Overall competency (Autonomy)	Unable to complete entire task, inefficient effort	Able to complete task safely even if task is slightly challenging	Able to complete task in spite of challenging case and can resolve complications (bleeding, leakage)

### Statistical analysis

Statistical analysis was performed with Sigmaplot 13.0 software (Systat Software, Inc., San José, CA, USA, 2014). The two-way ANOVA test was used to assess intra- and intergroup differences followed by the Holm–Sidak test. Data are presented as means ± SEM, *P* < 0.05.

## Results

**Fig. 3:**
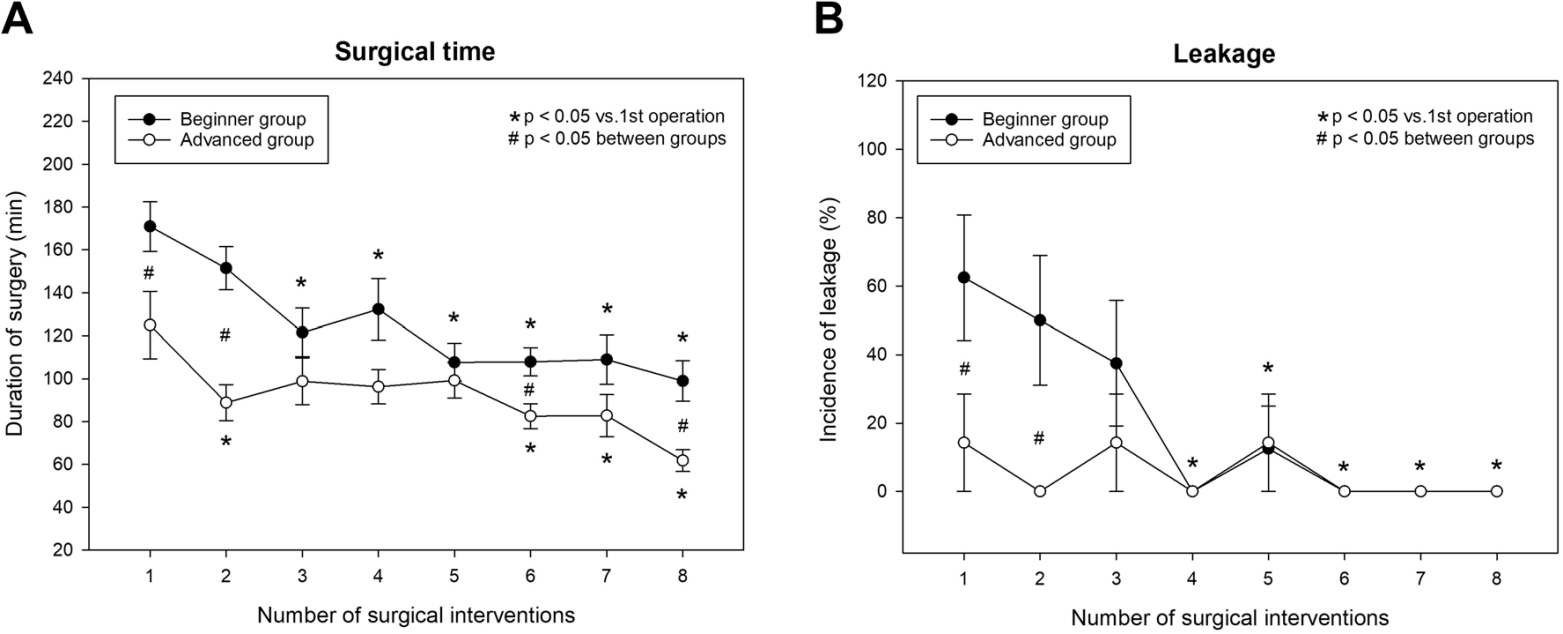
Time course of change in operation time, **A** and in anastomosis quality (incidence of leakage), **B** in the beginner and advanced groups

All of the participants succeeded in completing all eight test operations; 120 surgical interventions were, therefore, included in the analysis. The duration of the operations was uniformly longer in the beginner group than in the advanced group (Fig. 3A). This parameter showed a continuous decrease in both groups. In the beginner group, the operative time fell from 170.9 ± 11.6 to 107.1 ± 11.4 min (37.4%) versus a drop from 124.9 ± 15.6 to 61.8 ± 5.1 min (a 50.5% decrease) in the advanced group. Nevertheless, a significant difference persisted even during the sixth and eighth operations.

The leakage incidence was significantly higher in the beginner group in the first two interventions, but both groups showed similar results thereafter (Fig. 3B).

**Fig. 4:**
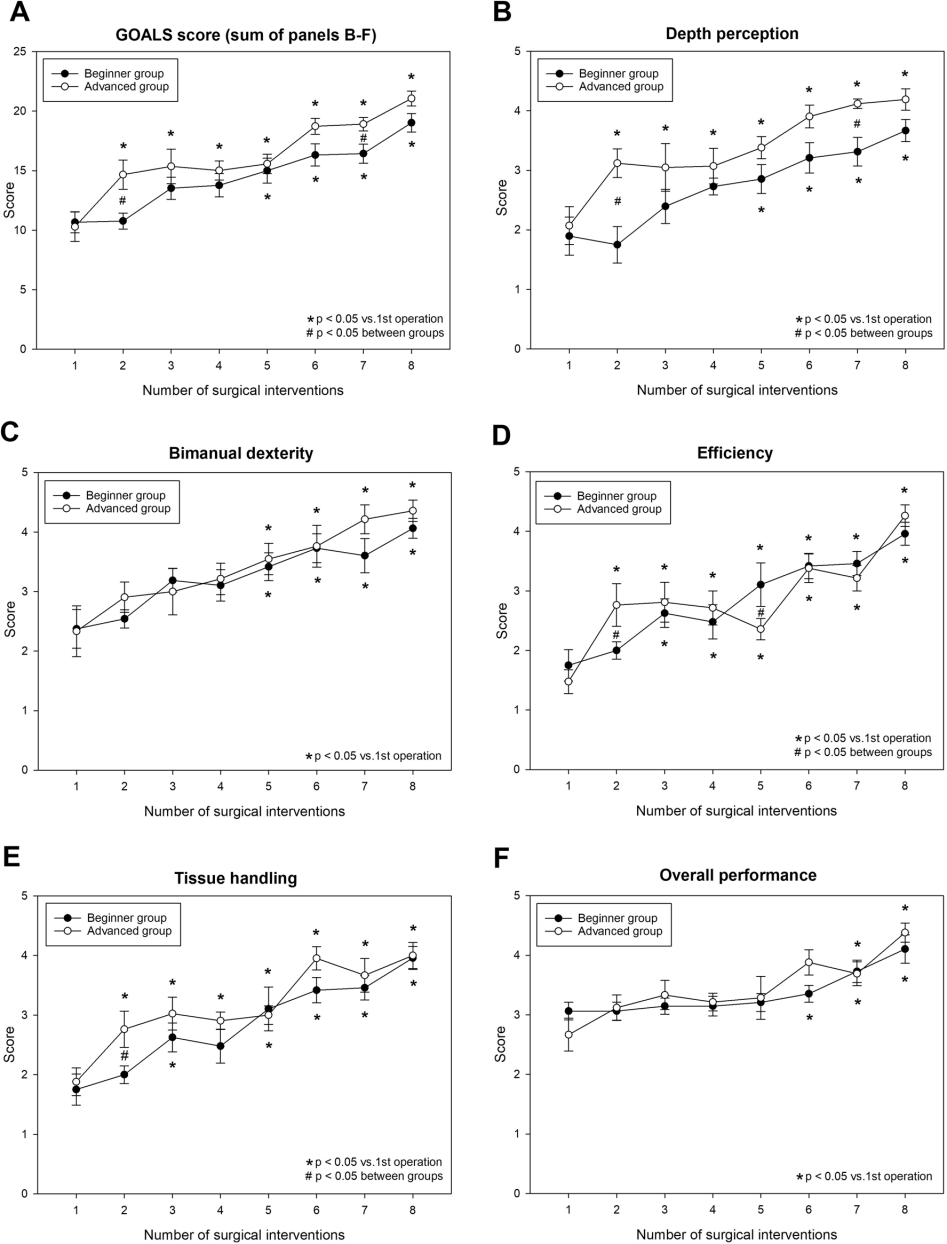
Average GOALS score (**A**), depth perception score (**B**), bimanual dexterity score (**C**), efficiency score (**D**), tissue handling score (**E**) and overall performance score in the beginner and advanced groups

Even though the expert evaluation (GOALS) score showed higher values for the advanced group (Fig. 4), it displayed a similar trend of improvement during the learning process for both groups. The difference only reached statistical significance at a few time points (i.e. at the second and seventh time points) of the study. In the beginner group, this positive progress with training started later than in the advanced group (being more evident in the second part of the study) (Fig. 4A). The early difference (seen at operation #2) between the study groups in the GOALS score was associated with inter-group differences in depth perception, tissue handling and efficiency scores (Fig. 4B, D and E), while the later difference in the GOALS score (seen at the seventh time point) resulted from a difference in depth perception (Fig. 4B). Bimanual dexterity and overall performance showed similar values during the entire study period in both groups.

**Fig. 5:**
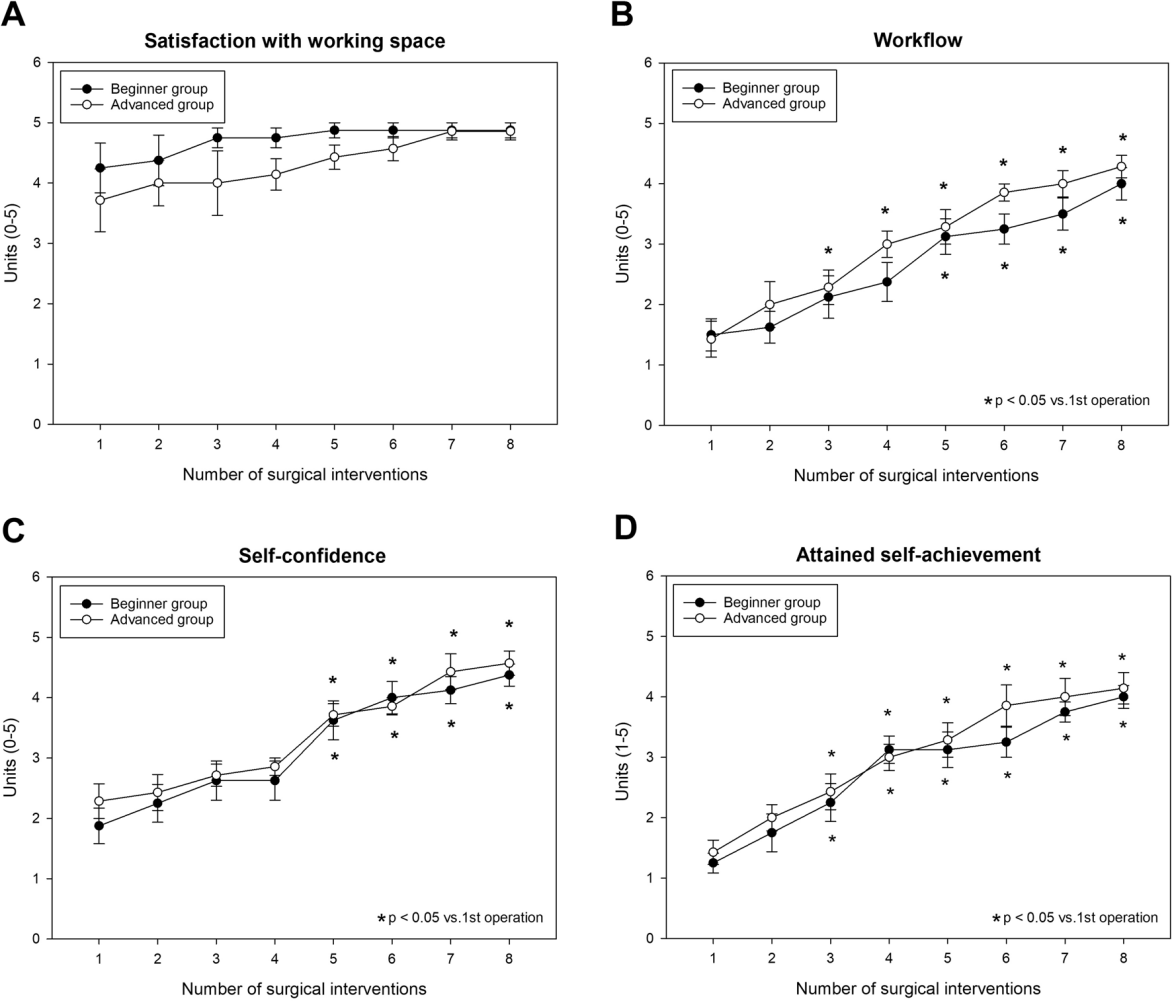
Participant feedback. Working space (**A**), workflow (**B**), self-confidence (**C**) and self-achievement (**D**)

Participant feedback forms reflect a nearly maximum satisfaction score with the size of the available workspace during the entire study period, whereas self-reflective parameters (workflow, self-confidence and self-achievement) showed gradual improvements with no significant differences between the groups (Fig. 5A−D).

## Discussion

Laparoscopic duodenal atresia repair is still one of the most challenging tasks in pediatric surgery [19, 20] requiring advanced laparoscopic abilities, including meticulous dissection and laparoscopic intracorporal knotting skills [20–22]. Performance, however, greatly improves over time due to the increased number of repetitions in clinical settings [16]. In general, at least 10–20 h of dry-lab training and a minimum of ten hours of animal model-based training have been recommended by the European Society of Pediatric Endoscopic Surgeons guidelines to gain expertise before performing basic laparoscopic human surgeries [23]. Repetition of interventions is also indispensable toward improved performance as proven by various preclinical and clinical studies (e.g. duodenal atresia and inguinal hernia) [16, 24], and it is particularly important in infants due to the small dimensions of the operative field. The size of the animals used in our model corresponds to the size of premature neonates who are often subjects of this rather challenging laparoscopic duodenal atresia operation. The feasibility of another rabbit model of laparoscopic duodenal atresia repair (gastro-duodenostomy) was also tested elsewhere, but it was only based on a single surgical intervention [13]. Here, characterization of the model was based on an analysis of individual learning curves during eight operations. Furthermore, the performance of the two trainee groups was compared with the aim of showing threshold expertise for the same procedure in clinical practice. Apart from providing a novel training model, probably another novelty of our present study is that the analysis was based on simultaneous consideration of multiple perspectives.

Surgery time is one of the most objective and easily accessible indices of performance [6, 12, 13, 25, 26], which was found to be similar to those in clinical practice for laparoscopic diamond-shaped anastomosis [22]. Owing to a standardized, structured MISTELS-based training in our study, the beginner group completed the tasks more slowly than the clinically experienced advanced group only at certain stages of the study. However, probably the most important and clinically most relevant measure of technical performance is the success of the intervention [13, 22] (here, anastomosis quality refers to the passage and water tightness of an anastomosis). This binary parameter is specific to the actual model. The fact that this desired outcome was reached relatively early in both groups shows (1) the efficacy of the dry-lab laparoscopic training of the beginner group and (2) the feasibility of the present in vivo rabbit model.

We also used other methods enjoying the advantage of including several aspects of surgical performance when challenging laparoscopic tasks are evaluated. Validated tools were used for the open surgical interventions (e.g. the Objective Structured Assessment of Technical Skills-OSATS) [27]; however, this is usually less applicable in minimally invasive interventions. For dry-lab laparoscopic training sessions, MISTELS and Laparoscopic Suturing Competency Assessment Tool scores are probably more adequate tools [15, 28], while the GOALS score appears to be an appropriate approach for both basic and complex interventions [29]. It enables assessors not only to classify surgeons based on their technical performance (detailed in the Methods section) [17, 25], but also to compare advancement in different training groups [12, 30]. We observed significant improvement in both groups as regards the GOALS score, but a statistically significant difference between study groups was only observed at a few stages of the learning process (and appeared only in terms of a few domains of the GOALS score, e.g. depth perception, tissue handling and efficiency). This underlines the efficacy of standardized laboratory (e.g. MISTELS) training enabling beginners to show similar results to those of more experienced colleagues during the test operation. Bansal et al. also used operation time and anastomosis quality to compare the performance of beginner and trained residents after laparoscopic training using five test operations in an *ex vivo* model of gastrojejunostomy. In their model, gradual improvements were found in all parameters, with minor and gradually vanishing initial differences between the two groups over time [25].

When the transferability of lab training findings to clinical situations is considered, the GOALS score may also represent a good tool to assess the efficacy of laparoscopy training [29–31]. In another study by Bansal et al., a five-day (wet-lab) laparoscopic training program for cholecystectomy resulted in marked differences in clinical performance of the same operation [30]. During their human “test operation” on a single occasion, the GOALS score (and each domain of the GOALS score) appeared to be significantly higher (together with other indices of improvement examined, i.e. surgery time and complication rate) in the trained group. In our study, international participants were recruited; therefore, a clinical “test operation” could not be conducted to assess the transferability of our findings, but this could be a highly important aspect of future studies.

Interestingly, certain trials found no significant improvement in participant performance at a certain stage after repeatedly conducting the same type of laparoscopic procedure. The same was demonstrated by Fu B et al. with an in vivo pyeloplasty in a porcine model [11], where a stationary phase was reached in the learning process after the fifth operation. Surgery duration showed further improvement in our study, while leakage rate and GOALS score values indicated a lower degree of improvement between the fifth and eight surgeries. This suggests that a minimum number of five laboratory surgeries is definitely needed in the present diamond-shaped anastomosis model, but any further skill development should most probably be monitored under clinical conditions. Our findings are also supported by results from participant feedback forms which are regarded as important tools to gain insights into personal or self-assessment [2, 12, 13]. The feedback questionnaire used in our study showed that both groups found the task similarly challenging at different stages of the study and the values gradually increased in parallel with the improvement in technical skills (as indicated by the GOALS score). Self-reflective parameters (particularly workflow and self-confidence) also showed significant improvement as of the fifth surgery (as compared to the baseline).

Our study has certain limitations, however. First, this model only focuses on the technical aspects (i.e. suturing skills) of laparoscopic duodenal atresia surgery, and dissection of the atresia sites, for instance, was not included in the protocol. This issue could be important because a misconducted distal pouch (e.g. in the case of type C atresia) could represent a source of severe complications. Another limitation is the lack of long-term follow-up and monitoring of surgical outcomes; this could have been overcome by establishing a surviving model. Furthermore, study participants in the advanced group were not selected and tested by standard criteria, whereas initial (dry-lab) performance was only assessed in the beginner group.

## Conclusions

In summary, our observations suggest that standardizing repetitive preclinical laparoscopic training tasks and using an in vivo model specifically designed for pediatric surgical challenges represent useful learning tools for pediatric residents. In our study, the performance of participants undergoing standardized laparoscopic skill training reached the levels of experienced, but non-uniformly trained residents during repetitive in vivo practice. We conclude that the diamond-shaped anastomosis model used on rabbits is particularly suited to simulating similar surgical interventions on humans. The same model or similarly complex methods may also be used for examination purposes before pediatric laparoscopic interventions. In our present study, sufficient anastomosis quality was achieved after the fifth surgery in this model with no further substantial improvements in objective skill assessments. Based on the minor further improvements after the fifth diamond-shaped anastomosis surgery in this animal setting, we assume that this number of interventions of this advanced operation is sufficient for laboratory training, presumably enabling residents to participate even in complex surgeries in clinical practice. Translation of the present results and further improvements in performance should probably be tested under clinical conditions where the importance of further skills (non-technical skills, e.g. decision-making expertise, stress-related factors and teamwork) can also be taken into consideration.

## Abbreviations

GOALS: Global Operative Assessment of Laparoscopic Skills
MISTELS: McGill Inanimate System for Training and Evaluation of Laparoscopic Skills
